# Negative Interactions and Feedback Regulations Are Required for Transient Cellular Response

**DOI:** 10.1038/srep03718

**Published:** 2014-01-16

**Authors:** Mohammad Mobashir, Thati Madhusudhan, Berend Isermann, Tilo Beyer, Burkhart Schraven

**Affiliations:** 1 Institute of Molecular and Clinical Immunology, Otto-von-Guericke University, 39120, Magdeburg, Germany; 2 Institute of Clinical Chemistry and Pathobiochemistry, Otto-von-Guericke University, 39120, Magdeburg, Germany; 3 Department of Immune Control, Helmholtz Centre for Infectious Disease (HZI), Inhoffenstrasse 7, 38124 Braunschweig, Germany

## Abstract

Signal transduction is a process required to conduct information from a receptor to the nucleus. This process is vital for the control of cellular function and fate. The dynamics of signaling activation and inhibition determine processes such as apoptosis, proliferation, and differentiation. Thus, it is important to understand the factors modulating transient and sustained response. To address this question, by applying mathematical approach we have studied the factors which can alter the activation nature of downstream signaling molecules. The factors which we have investigated are loops (feed forward and feedback loops), cross-talk of signal transduction pathways, and the change in the concentration of the signaling molecules. Based on our results we conclude that among these factors feedback loop and the cross-talks which directly inhibit the target protein dominantly controls the transient cellular response.

Cells transmit and receive information through signal transduction process by controling the dynamics of the intracellullar signaling molecules (SMs)^1^,^2^,^3^,^4^,^5^. The temporal dynamics of SMs plays critical roles in making cellular decisions^5^,^6^,^7^,^8^,^9^. For example, PC-12 cells after NGF treatment causes sustained Erk activation leads to differentiation of the PC-12 cells, whereas transient Erk activation induces proliferation^10^. From the previous published data^11^,^12^,^13^,^14^,^15^, it appears that there are many important diseases which arise due to aberrations in the signal transduction process. The critical point is the cellular response duration (nature) which seems to be directly linked to the cell-fate decision^10^,^16^,^17^,^18^,^19^,^20^. Based on the nature of the cellular response (transient or sustained or partially adapted), the cells undergo apoptosis, proliferation, or differentiation^10^,^18^,^19^. Thus, it is an important step in signal transduction process to understand the interaction of the signaling pathways resulting in transient or sustained cellular response.

In the past, many research groups have focused on the signal transduction pathways and investigated different factors which may play critical roles in controling the cellular response nature and finally the cell-fate (or cell-fate decision)^9^,^21^,^22^,^23^. The factors which have been investigated so far are the rate of reactions^24^,^25^, network topology^25^, concentration of the SM^26^,^27^, feed forward loops (FFLs), feedback loops (FBLs)^21^,^22^, or the cross-talk of the signal transduction pathways^28^,^29^,^30^,^31^,^32^,^33^,^34^,^35^.

In biological systems, mainly four different types of cross-talks ((i) concomitant signaling, (ii) collaborative signaling, (iii) direct signaling, and (iv) amplification of signaling), have been reported^31^. Unlike to these previous works, we have started the investigation of a minimal cascade to the complex signaling regulation by adding all the possible interactions in one model.

Some of the FBLs^22^, FFLs^36^, and cross-talks^28^,^29^,^31^,^37^,^38^,^39^ have been investigated in biological signaling. In addition to these previously studied possible regulations, we have included more possible FFLs (both positive and negative), FBLs (both positive and negative), the combination of FFLs and FBLs, and increased more cross-talk possibilities (both the cross-interactions between the cascades i.e., inhibition and activation) between the linear cascades in one model and investigated their impact in controling the cellular response nature. From our results, we conclude that FBL and cross-talk plays critical role in determining transient cellular response. This model will help to understand the cellular response nature, to further reveal the new interactions based on the desired output response, and to perturb the output response by targeting the specific SM.

## Results

**Figure 1:**
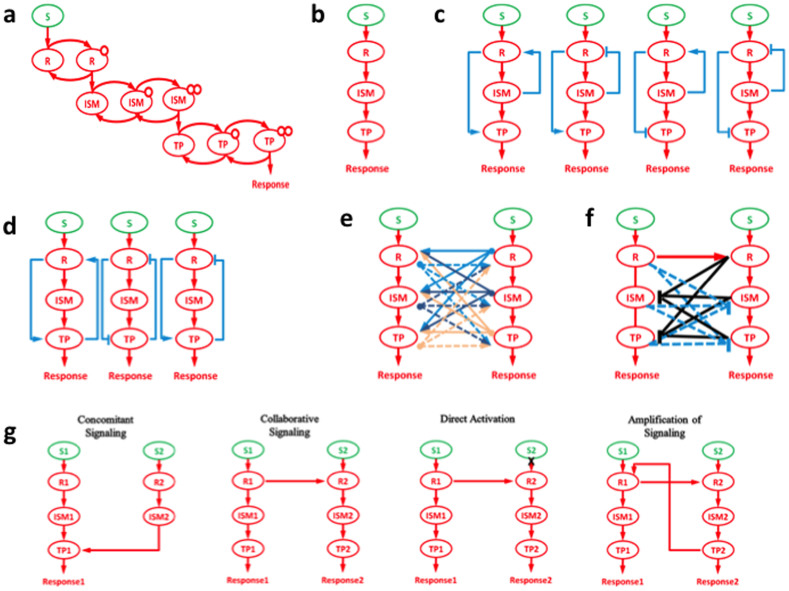
Signaling cascade and its regulations. S, R, ISM, and TP stand for input signal, receptor, intracellular signaling molecule, and target protein, respectively. (a) A typical linear signaling cascade where R after detecting input signal S becomes active (goes to post-translational modification (e.g., phosphorylated)), active R activates ISM (single or double phosphorylation) and finally active ISM activates TP (single or double phosphorylation), (b) its simplified form, and (c) and (d) represents possible feed forward and feedback regulation (both positive (arrow) and negative regulation (blocked line)). (e) and (f) represent the cross-talks (arrows – activation and lines with blocked end -- inhibition) between signal transduction pathways (cascades). (g) cross-talks known in biological signal transduction^31^.

As mentioned in the previous section, some of the FBLs, FFLs, and cross-talks have been investigated in biological signaling. In addition to these previously studied possible regulations, we have included more possible FFLs (both positive and negative), FBLs (both positive and negative), the combination of FFLs and FBLs, and increased more cross-talk possibilities (both the cross-interactions between the cascades i.e., inhibition and activation) between the linear cascades in one model (Figure 1 a, b, c, d, e, f, and g) and investigated their impact in controling the cellular response nature. The major difference between the previous works and our work is the investigation of the combinations of different kinds of FFLs and FBLs and more cross-interactions between the signaling cascades in the presence and absence of FFLs and FBLs than the four positive cross-talks (Figure 1g) reported by Ivaska J and Heino J^28^,^29^,^30^,^31^,^34^,^40^,^41^,^42^,^43^,^44^,^45^. In this model, the complex signaling networks have been simplified and represented as receptor level (R), intracellular signaling level (ISM), and target level (TP). So that the effect of different kinds of interactions at different levels on the final cellular response nature can be studied.

### A linear cascade always produces sustained cellular response

Here, we have investigated the kinetics of the signaling molecules for linear cascade (a cascade without feed forward loop, feedback loop, and cross-talk between a pair of linear cascades) and linear cascades with feed forward loop and feedback loop (Figure 1 a, b, c, and d).

**Figure 2:**
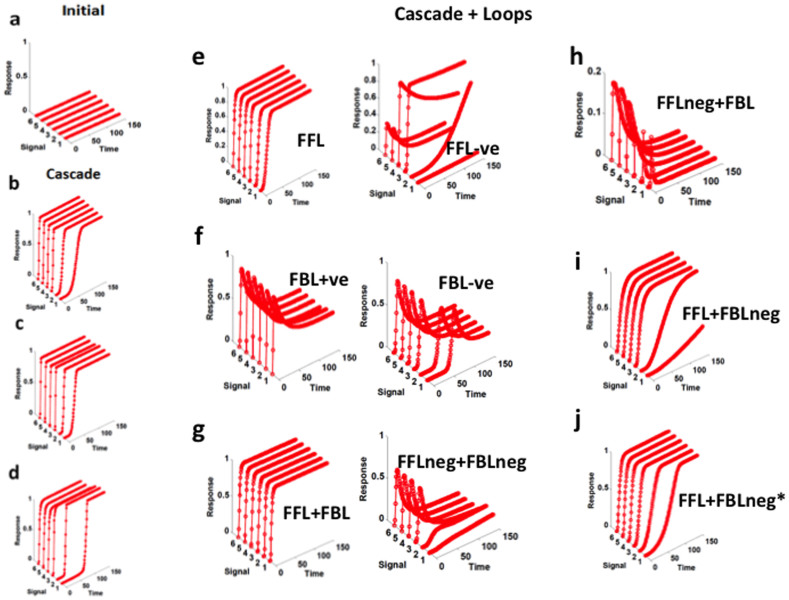
Response kinetics (normalized value) of the signaling cascade. (a) Initially, kinetics of all the signaling cascade with or without additional regulation (e.g., FFL, FBL, or cross-talks) stays close to zero (*k_par_* are generated randomly between 0.001 and 0.1). (b) kinetics of the fully evolved signaling cascade (the concentration of R, ISM, and TP are fixed and equal i.e., 10 μl, during the evolutionary period the signaling cascades were allowed to adapt new *k_par_* (between 0.1 and 100) values to improve the kinetic response). (c) kinetics of the fully evolved signaling cascade (the concentration of R, ISM, and TP are fixed and unequal i.e., 10 μl, 5 μl, and 1 μl, respectively, during the evolutionary period the signaling cascades were allowed to adapt new *k_par_* (between 0.1 and 100) values to improve the kinetic response). (d) kinetics of the fully evolved signaling cascade (initially the concentration of R, ISM, and TP are fixed and equal i.e., 10 μl, during the evolutionary period the signaling cascades were allowed to adapt new *k_par_* (between 0.1 and 100) values and change in the concentration of R, ISM, and TP to improve the kinetic response). (e) kinetics of the fully evolved signaling cascade in the presence of FFL (the concentration of R, ISM, and TP are fixed and unequal i.e., 10 μl, 5 μl, and 1 μl, respectively, during the evolutionary period the signaling cascades were allowed to adapt new *k_par_* (between 0.1 and 100) values to improve the kinetic response). (f) kinetics of the fully evolved signaling cascade in the presence of FBL, (g) FFL and FBL (both positive and negative), (h) negative FFL and FBL, (i) FFL and negative FBL, and (j) FFL and negative FBL from ISM to R (the concentration of R, ISM, and TP are fixed and unequal i.e., 10 μl, 5 μl, and 1 μl, respectively, during the evolutionary period the signaling cascades were allowed to adapt new *k_par_* (between 0.1 and 100) values to improve the kinetic response).

For this purpose, we have generated linear cascades with different sets of kinetic parameters (*k_par_*). In case of signaling networks, the unit of *k_par_* can be second^−1^ or minute^−1^ 46. It is known that in general, the signal transduction process is fast and can function on the timescale of seconds to minutes^47^. Throughout our work, we have written time instead of second or minute. Initially, *k_par_* were randomly generated between 0.001 to 0.1. So, all the cascades have response kinetics close to zero (Figure 2a). Then, we have applied an evolutionary algorithm (EA)^24^,^48^ to evolve the cascades. During the evolutionary period, we allowed the change in *k_par_* and the concentration level of SMs. In this period, the signaling cascade adapts the improved kinetic parameters to produce better response.

After analyzing the kinetics of the evolved networks, we observe that in a linear signaling cascade (without any FFL/FBL), the change in the kinetic parameters or the concentration does not produce any transient response (Figure 2b, c, and d). Increase in the concentration (SMs) or the kinetic parameter values leads to improved sustained response (Figure 2b, c, and d).

Addition of a positive FFL in a signaling cascade (Figure 1c) does not change the cascade response and it remains sustained (Figure 2e, left) while the addition of a negative FFL disturbs the output response. The addition of a negative FFL produces mixed response either as transient, or sustained, or complete blocking of the response (Figure 2e, right) which means the activation pattern is not robust. Addition of FBL (positive or negative) leads to transient response (Figure 2f). Presence of one positive FFL and a positive FBL leads to sustained response (Figure 2g, left), presence of one negative FFL and a negative FBL and one negative FFL and a positive FBL leads to transient response (Figure 2 g and h), and presence of one positive FFL and a negative FBL does not change the sustained response to transient nature (Figure 2i). These FFL (positive or negative) and FBL (positive or negative) are from R to TP or TP to R (Figure 1d). When we apply the FBL (positive or negative) and/or FFL (positive or negative) from R to ISM or ISM to R in a cascade, we always observe sustained output response (Figure 2j).

### Concomitant inhibition between cascades dominantly produce transient response

**Figure 3:**
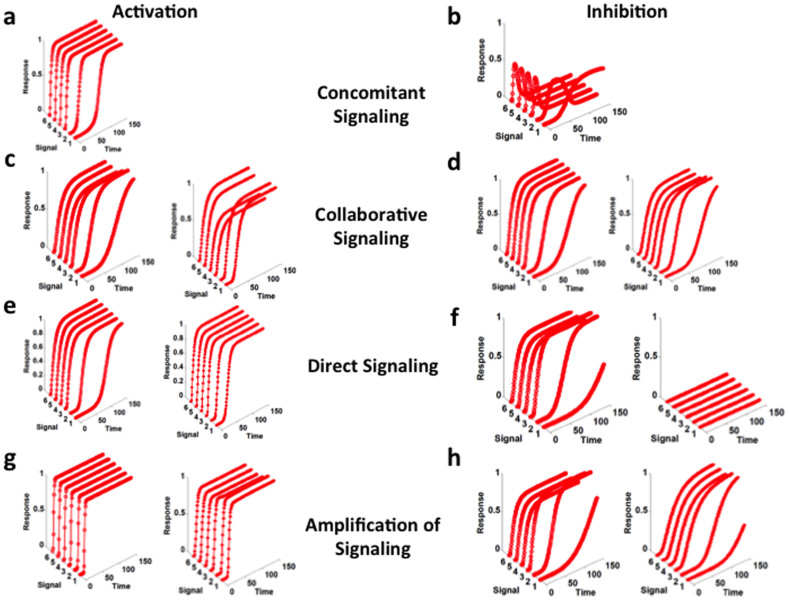
Kinetics of output response in case of cross-talk between the signaling cascades. (a) activation concomitant signaling, (b) inhibitory concomitant signaling, (c) activation type collaborative signaling, (d) inhibition type collaborative signaling, (e) direct signaling – activation, (f) direct signaling – inhibition, (g) amplification of signaling – activation, and (h) amplification of signaling – inhibition. In figure c, d, e, f, g, and h, left side figure represents the kinetics of the output response of cascade 1 and right side figure represents the kinetics of output response of cascade 2.

After analyzing the kinetics of signaling cascade response, we investigated the change in the kinetics of the TP of the signaling cascade in the presence of different kinds of cross-talks known from biological system^31^. We have investigated their inhibitory forms (in biological cross-talks the links between the cascades are activation) also for all the four cross-talks. We found that concomitant signaling (activation link between two cascade) leads to sustained response (Figure 3a) and its inhibitory form produces transient response (Figure 3b). While all the three other kinds of cross-talks (collaborative, direct, and signal amplification) between the cascades help in producing stable sustained response (Figure 3 c, d, e, f, g, and h) irrespective the nature (activation or inhibition) of the links between the cascade. In case of direct signaling, inhibitory interaction between the two cascades leads to only one output response in cascade 1 and complete blockage of the output response of cascade 2 (Figure 3f) because here input signal (S_2_) is blocked.

### Increase in the number of inhibitory links leads to transient response or complete blockage of the output response

Finally, we have investigated the effect of all the possible interactions (FFL, FBL, and cross-talks) in a single model. Here, we have two linear cascades in parallel without any cross-interaction. We have generated 200 sets of parallel cascades and evolved them in parallel until 100 generations by allowing the rate of reactions (*k_par_*) to change during evolutionary period to adapt new *k_par_* in order to produce improved kinetic response. After 100 generations, all the new interactions were added one-by-one in a linear signaling cascade (Figure 1a) in each generation. In this work, first we have started addition of negative interactions between two cascades, then FFL and FBL, and finally the positive interactions between cascades.

**Figure 4:**
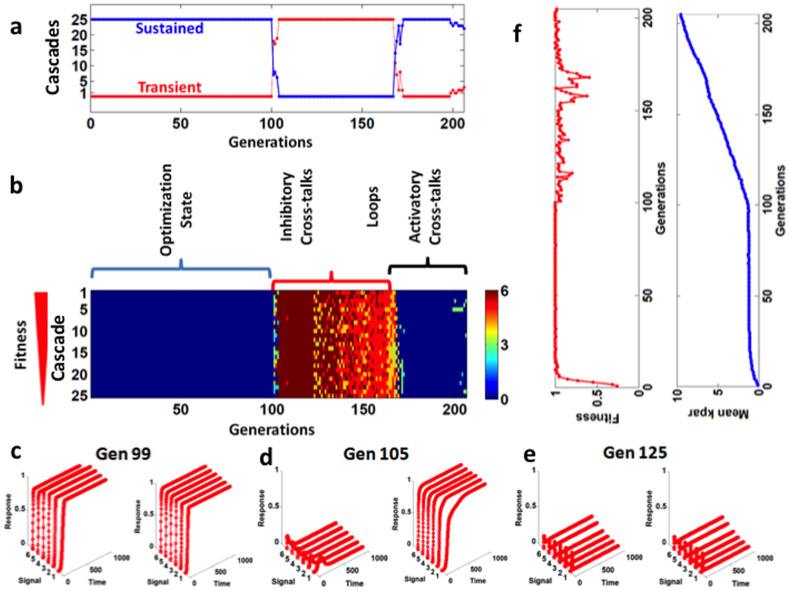
Change in response kinetics of the signaling cascade from simple cascade (without FFL, FBL, and cross-talk) to complex cascade (with FFL, FBL, and cross-talks). (a) Total number signaling cascades with transient and sustained response among the best 25 signaling cascades. (b) As in our model for each cascade we have six input signals (of different strength) so we have six output response. Here, we show total number of response (transient and/or sustained) in each cascade. (c), (d), and (e) show the kinetics of the output response (cascade 1 (left side figure) and cascade 2 (right side figure)) in generation 99, 105, and 125, respectively. (f) Mean fitness (left side) and the mean of *k_par_* (right side) of cascades (best cascades).

We observe that all the minimal cascades produce sustained output response for all the six different (strength) input signals (Figure 4 a and b). In contrast, addition of new inhibitory interactions between the two cascades, FFL, and FBL leads to transient response which can be seen between generation 100 and 165 in Figure 4 a and b. The response nature has been shown in Figure 4c (for a linear cascade – where both the cascades produce sustained output response before generation 100 (left – pathway 1 and right – pathway 2)). Since, in the beginning we add the interactions through which pathway 2 inhibits pathway 1 so the output response of pathway 1 is transient and pathway 2 remains sustained (Figure 4d). When we add the interactions (inhibitory) between both the pathways then both the pathways produce transient response or completely block the output response of both the pathways (Figure 4e). Addition of positive interactions between the cascades lead to the sustained output response which can be seen in Figure 4 a and b after generation 165 and the kinetics of the output appears similar to Figure 4c. As far as the fitness of the cascades is concerned, as long as the cascades are free from additional interactions, the fitness remain stable and stays at maximum (Figure 4f (left)) because the kinetics of all the cascades for all the input signals easily crosses the threshold level and remain sustained. While addition of new inhibitory interactions between the cascades and the FFL and FBL shows fluctuation in the fitness because the output response becomes either transient or does not crosses the threshold. We further investigated the change in the *k_par_*. In linear cascade which has comparatively less number of reactions so the mean of the *k_par_* is comparatively lower than the cascade with new interactions and the addition of new interactions in each generation leads to the gradual increase in the *k_par_* (Figure 4f (right)). So, we conclude that the irrespective the response nature (sustained or transient) the *k_par_* increases but it does not affect the cellular response nature.

### Bistable behavior

**Figure 5:**
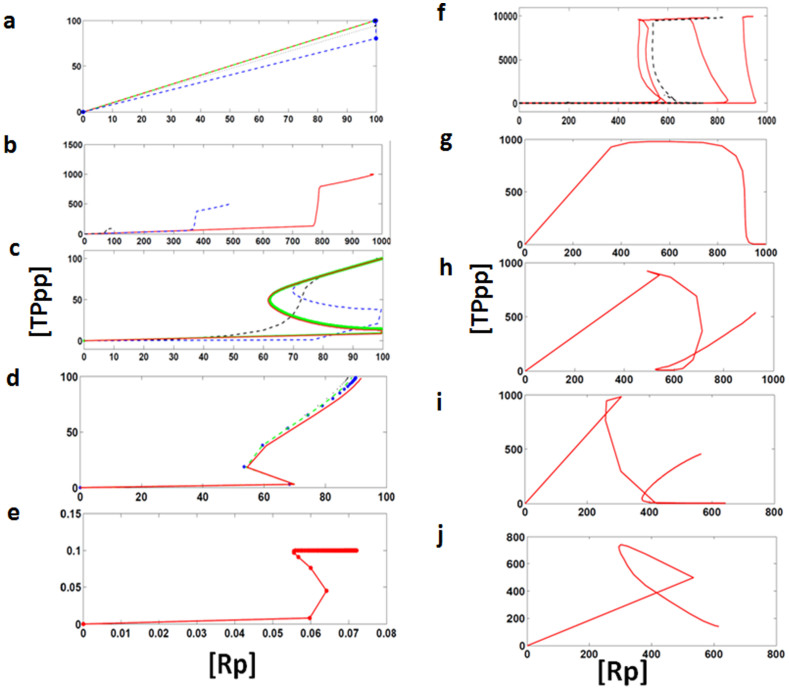
Dose-response curve. x-axis represents total concentration of activated receptor ([Rp]) and y-axis represents total concentration of output response ([TPpp]). (a) Red, blue, and black curves are representing dose-response curve for three different sets of kinetic parameters. (b) For one fixed set of kinetic parameters (randomly selected from the evolved cascades), the total output response have been plotted for three different concentration of R and TP. Red, blue, and black curves are representing dose-response curve for three different concentration of R and TP is 1000 μl, 500 μl, and 100 μl, respectively (positive FBL). (c) signal-response curve in the presence of a positive FBL and (d) signal-response curve in the presence of a negative FBL. Black curve represents the dose-response curve plotted for a set of kinetic parameter (one of the evolved cascade randomly selected) then we increase the kinetic parameter values which are involved in positive FBL blue curve (*k_par_* updated with the values between 10 and 100), green curve when positive FBL *k_par_* is between 100 and 500), and red curve where the positive FBL *k_par_* is between 500 and 1000. (e) Signal-response curve in the presence of a FFL and a FBL, (f) signal-response curve in the presence of a negative FFL and a negative FBL. (g), (h), and (i) are the signal-response curve in the presence of inhibitory cross-interactions between the cascades and (j) signal-reponse curve in the presence of activation links between the two cascades.

After analyzing the kinetics of the output response, we have also analyzed the bistable behavior^49^,^50^,^51^ for the output response (TPpp) of linear cascade (without FFL, FBL, or cross-talk) and the cascade with FFL, FBL, and cross-talk. We found that in case of a linear cascade and a cascade with positive FFL, and a linear cascade with negative FFL, the response is linear (Figure 5a). We observed bistable behavior for a cascade with FBL (positive/negative) and combination of FFL (positive/negative) and FBL (positive/negative) (Figure 5 b, c, d, e, and f). In case of negative cross-interaction between cascades with few exceptions (Figure 5g) the multistable response behavior appears to be dominant (most of the successful evolved networks show multiple stable states) (Figure 5 h, I, and j).

## Discussion

In this study, we have investigated the change in the output response nature (sustained or transient) of the signaling cascade in the presence and absence of the FFL, FBL, and cross-talks between two cascades. The cascade which we have used here, is similar to the MAPK cascade^1^. Based on our data, we propose that transient signaling responses result from FBL and/or negative cross-interactions between signaling cascades. If the concentration of the TP is lower than the concentration of the R and ISM, and either FBL or negative cross-talks are present then all the cascade produce consistently transient output response. Irrespective of the concentration of the signaling molecules, FFL and all the positive interactions (cross-talks) between the cascades lead to stable and sustained output response.

The evolved networks in a stationary population show stable activation pattern against the change in kinetic parameters for both signaling cascades (until generation 100) and addition of the positive interactions between the signaling cascades (after generation 165 onwards) and the output response as sustained response. This suggests that this type of response is the generic cellular behavior when the presence of a signal is sufficient information for a cell. While in the presence of inhibitory interactions between the signaling cascades and the cascade with FBL and the simultaneous presence of FBL and FFL, the kinetics of the output response is always transient (if the concentration of the TP is less than R, and ISM). The fitness of the cascades fluctuates significantly. This suggests the transient response as the generic solution. If the concentration of R, ISM, and TP is equal then the cascades with inhibitory cross-talks and the cascade with FBL or with combination of FFL and FBL also produces the transient response but not all the cascades (with the exceptions of few cascades having sustained response).

From previous works^21^,^22^,^23^,^31^,^52^,^53^, some interesting facts about the effect of variation in the concentration of SMs, FBL, FFL, and cross-talk of signaling pathways are known. Here, they have investigated the role of change in the concentration of an individual molecule and not investigated in comparison to the other molecules involved in signaling. The FBL, FFL, or cross-talk of pathways have been investigated individually and not in combination of FFL and FBL or cross-talk.

Most of the complex and/or common diseases such as cancer, diabetes, obesity, and asthma are caused by defects in multiple genes and pathways. So, it is not surprising that the current one-target-one-compound approach in drug discovery and development has failed to deliver as many efficacious medicines as expected in the post-genomic era^15^,^37^,^54^. In order to understand such complex diseases and find therapeutic solution, it appears to be promising point to understand the signal transduction process from a simple linear cascade to a complex regulatory mechanism (a linear cascade with different loops and the cross-interactions of the cascade) of signaling network. By applying this approach, we can selectively target the signaling molecules to get the desired output response and will help to target multiple signaling molecules.

The advantage of our model is that it will not only help to understand the effect of the variation in the concentration of the receptor molecules but also help to understand the impact of the concentration of other signaling molecules (such as intermediate and effector molecules) involved in the signal transduction and will give an insight of the different additional regulations such as FFL, FBL, and cross-talk. These models can only be applied to those systems which are known to have such behavior, but often the exact behavior of the STNs is not known. Therefore, the creation of a fitness function that encodes the task that a cell solves under certain experimental conditions, may be more beneficial in determining possible and likely behavior of the underlying signaling cascades.

## Conclusion

Based on our data, we conclude that the transient response is controlled by the FBL and the negative cross-interactions between the cascades. If the concentration of the TP is lower than the concentration of the R and ISM, and either FBL or negative cross-talks are present then all the cascade produce consistently transient output response. Irrespective of the concentration of the signaling molecules, FFL and all the positive interactions (cross-talks) between the cascades lead to stable and sustained output response.

## Methods

### Model

We have set up a signaling cascade which function in the similar way as MAPK signaling cascade works (Figure S1). This signaling cascade is divided into several different levels of signaling such as receptor level (represented as R), intracellular signaling level (as ISM), and the target level represented as TP (the target proteins are those proteins which communicate the information to the nucleus in the form of the output response). Then, we have added different kinds of loops and the cross-interactions (cross-talks) between two signaling cascades at different levels of signaling. In the next step, we have created mass-action kinetic model by using the ordinary differential equations (ODEs) for all the molecules including the complexes formed as result of chemical reaction. In simplified form, the temporal change in the concentration of the signaling components (SMs including the complexes formed) can be represented as: 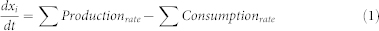


After the calculation of the kinetics of the all the molecules (1), we have calculated the fitness of all the cascades. For all the calculations, we have used six different input signals (*n_s_* = 6). The six different input signals (different in strength) were 0.0001, 0.001, 0.01, 0.1, 1, and 10. For each input signal (*n*), the kinetics of TP is tested whether it exceeds the threshold level (*TL*). The threshold (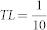 of the initial concentration of TP) is defined to be the relative fraction of double phosphorylated form of TP. In case of cascade with or without FBL and FFL, If this double phosphorylated form of TP crosses the threshold level at any time point then the cascade is assigned a value 1.0 called fitness factor (*F_factor_*_1_) and *F_factor_*_2_ = 0. In case of cross-talk of two cascades, in the similar way we check the kinetics of the double phosphorylated form of TP of both the cascades, if the kinetics of cascade 1 crosses the threshold at any time point then we assign fitness factor (*F_factor_*_1_) a value of 0.5 and if the kinetics of cascade 2 crosses the threshold at any time point then we assign fitness factor (*F_factor_*_2_) a value of 0.5 for cascade 2. After evaluating the kinetics for each cascade for all the six input signals (different in strength), we calculate the fitness (*F*) by taking the mean of the fitness factors (*F_factor_*_1_ *and* *F_factor_*_2_) which can be represented as: 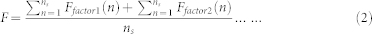


### Work flow

We have created a set of cascades (total number of cascades 200) with randomly generated kinetic parameters (*k_par_*) between 0.001 and 0.1. EA^24^,^55^ has been applied to evolve the signaling cascades. For each cascade, we have *F*. After calculating *F* of all the cascades (2), we select the best 50 cascades (successful cascades) based on higher *F* values. In order to improve the response kinetics, these successful cascades are allowed to adapt new *k_par_*. Four copies of all these 50 cascades with updated *k_par_* are created to keep the total number of cascades equal in each iteration. All these processes are repeated for 200 iterations. Each iteration is called as a generation. Sets of ODEs have been solved with MATLAB 7.9.0.

### Additional information

MM and TB were supported in part by grants from the German Ministry of Education and Research (BMBF FORSYS program) [0313922], the State of Sachsen-Anhalt (Dynamic Systems) [XD3639HP/0306], and the European Union (SYBILLA) [HEALTH-F4-2008-201106]. No additional external funding received for this study. The funders had no role in study design, data collection and analysis, decision to publish, or preparation of the manuscript. The authors declare no potential conflict of interest.

## Author Contributions

M.M. carried out research design and performed study, data analysis, and wrote main manuscript text and prepared figures. M.T. helped in designing research. B.I. helped in designing research and contributed in manuscript writing. T.B. and B.S. designed research and contributed to write the manuscript. All authors read and approved the final manuscript.

## Supplementary Material

Supplementary Information S1: Minimal cascade and its reaction details

## References

[b1] Hornberg J. J. *et al.* Control of MAPK signalling: from complexity to what really matters. Oncogene 24, 5533–5542 (2005). 16007170 10.1038/sj.onc.1208817

[b2] Chaudhri V. K., Kumar D., Misra M., Dua R. & Rao K. V. S. Integration of a phosphatase cascade with the mitogen-activated protein kinase pathway provides for a novel signal processing function. J Biol Chem 285, 1296–1310 (2010). 19897477 10.1074/jbc.M109.055863 PMC2801257

[b3] Aoki K., Yamadac M., Kunidac K., Yasudaa S. & Matsudaa M. Processive phosphorylation of ERK MAP kinase in mammalian cells. Proc Natl Acad Sci USA 108, 12675–12680 (2011). 21768338 10.1073/pnas.1104030108 PMC3150946

[b4] Ferrell J. E. & Bhatt R. R. Mechanistic studies of the dual phosphorylation of mitogen- activated protein kinase. J Biol Chem 272, 19008–19016 (1997). 9228083 10.1074/jbc.272.30.19008

[b5] Acuto O., Bartolo V. D. & Michel F. Tailoring T-cell receptor signals by proximal negative feedback mechanisms. Nat Rev Immunol 8, 699–712 (2008). 18728635 10.1038/nri2397

[b6] Purvis J. E. & Lahav G. Encoding and Decoding Cellular Information through Signaling Dynamics. Cell 152, 945–956 (2013). 23452846 10.1016/j.cell.2013.02.005 PMC3707615

[b7] Lahav G. *et al.* Dynamics of the p53-Mdm2 feedback loop in individual cells. Nat Genet 36, 147–150 (2004). 14730303 10.1038/ng1293

[b8] Ferrell J. E. Jr The biochemical basis of an all-or-none cell fate switch in xenopus oocytes. Science 280, 895–898 (1998). 9572732 10.1126/science.280.5365.895

[b9] Kubota H. *et al.* Temporal coding of insulin action through multiplexing of the AKT pathway. Mol Cell 46, 820–832 (2012). 22633957 10.1016/j.molcel.2012.04.018

[b10] Marshall C. J. Specificity of receptor tyrosine kinase review signaling: transient versus sustained extracellular signal-regulated kinase activation. Cell 80, 179–185 (1995). 7834738 10.1016/0092-8674(95)90401-8

[b11] Hanahan D. & Weinberg R. A. Hallmarks of cancer: the next generation. Cell 144, 646–674 (2011). 21376230 10.1016/j.cell.2011.02.013

[b12] Testa J. R. & Tsichlis P. N. AKT signaling in normal and malignant cells. Oncogene 24, 7391–7393 (2005). 16288285 10.1038/sj.onc.1209100

[b13] Altomare D. A. & Testa J. R. Perturbations of the AKT signaling pathway in human cancer. Oncogene 24, 7455–7464 (2005). 16288292 10.1038/sj.onc.1209085

[b14] Gunawardena J. Signals and systems: towards a systems biology of signal transduction. Proc. IEEE 96, 1386–1397 (2008).

[b15] Downward J. Targeting RAS signalling pathways in cancer therapy. Nat Rev Cancer 3, 11–22 (2003). 12509763 10.1038/nrc969

[b16] Purvis J. E. & Lahav G. Encoding and decoding cellular information through signaling dynamics. Cell 152, 945–956 (2013). 23452846 10.1016/j.cell.2013.02.005 PMC3707615

[b17] Kumar D., Srikanth R., Ahlfors H., Lahesmaa R. & Rao K. V. S. Capturing cell-fate decisions from the molecular signatures of a receptor-dependent signaling response. Mol Syst Biol 3, (2007). 10.1038/msb4100197 PMC2174630 18059445

[b18] Winstead C. J. & Weaver C. T. Dwelling on T cell fate decisions. Cell 153, 739–741 (2013). 23663773 10.1016/j.cell.2013.04.026

[b19] Tyson J. J. *et al.* Dynamic modelling of oestrogen signalling and cell fate in breast cancer cells. Nat Rev Cancer 11, 523–532 (2011). 21677677 10.1038/nrc3081 PMC3294292

[b20] Berg N. N., Puente L. G., Dawicki W. & Ostergaard H. L. Sustained TCR signaling is required for mitogen-activated protein kinase activation and degranulation by cytotoxic T lymphocytes. J Immunol 161, 2919–2924 (1998). 9743353

[b21] Loewer A. & Lahav G. Cellular conference call: external feedback affects cell-fate decisions. Cell 124, 1128–1130 (2006). 16564006 10.1016/j.cell.2006.03.010

[b22] Poltorak M. *et al.* TCR activation kinetics and feedback regulation in primary human T cells. Cell Commun Signal 11, 4 (2013). 23317458 10.1186/1478-811X-11-4 PMC3842781

[b23] Spencer S. L. & Sorger P. K. Measuring and modeling apoptosis in single cells. Cell 144, 926–939 (2011). 21414484 10.1016/j.cell.2011.03.002 PMC3087303

[b24] Mobashir M., Schraven B. & Beyer T. Simulated evolution of signal transduction networks. PLoS ONE 7, e50905 (2012). 23272078 10.1371/journal.pone.0050905 PMC3521023

[b25] Francois P. & Hakim V. Design of genetic networks with specified functions by evolution in silico. Proc Natl Acad Sci USA 101, 580–585 (2004). 14704282 10.1073/pnas.0304532101 PMC327190

[b26] Spencer S. L. & Sorger P. K. Measuring and modeling apoptosis in single cells. Cell 144, 926–939 (2011). 21414484 10.1016/j.cell.2011.03.002 PMC3087303

[b27] Spencer S. L., Gaudet S., Albeck J. G., Burke J. M. & Sorger P. K. Non-genetic origins of cell-to-cell variability in TRAIL-induced apoptosis. Nature 459, 428–432 (2009). 19363473 10.1038/nature08012 PMC2858974

[b28] McClean M. N., Mody A., Broach J. R. & Ramanathan S. Cross-talk and decision making in MAP kinase pathways. Nat Genet 39, 409–414 (2007). 17259986 10.1038/ng1957

[b29] Aksamitiene E., Kiyatkin A. & Kholodenko B. N. Cross-talk between mitogenic Ras/MAPK and survival PI3K/Akt pathways: a fine balance. Biochem Soc Trans 40, 139–146 (2012). 22260680 10.1042/BST20110609

[b30] Negishi H. *et al.* Cross-interference of RLR and TLR signaling pathways modulates antibacterial T cell responses. Nat Immunol 13, 659–666 (2012). 22610141 10.1038/ni.2307

[b31] Ivaska J. & Heino J. Cooperation Between Integrins and Growth Factor Receptors in Signaling and Endocytosis. Annu Rev Cell Dev Biol 27, 291–320 (2011). 21663443 10.1146/annurev-cellbio-092910-154017

[b32] Basak S. & Hoffmann A. Crosstalk via the NF-κB signaling system. Cytokine *&* Growth Factor Reviews 19, 187–197 (2008). 18515173 10.1016/j.cytogfr.2008.04.005 PMC2675004

[b33] Ogawa S. *et al.* Molecular determinants of crosstalk between nuclear receptors and Toll-like Receptors. Cell 122, 707–721 (2005). 16143103 10.1016/j.cell.2005.06.029 PMC1430687

[b34] Bezbradica J. S. & Medzhitov R. Integration of cytokine and heterologous receptor signaling pathways. Nat Immunol 10, 333–339 (2009). 19295629 10.1038/ni.1713

[b35] Fraser I. D. C. & Germain R. N. Navigating the network: signaling cross-talk in hematopoietic cells. Nat Immunol 10, 327–331 (2009). 19295628 10.1038/ni.1711 PMC2776706

[b36] Doncic A. & Skotheim J. M. Feedforward regulation ensures stability and rapid reversibility of a cellular state. Mol Cell 50, 856–868 (2013). 23685071 10.1016/j.molcel.2013.04.014 PMC3696412

[b37] Sands W. A., Copland M. & Wheadon H. Targeting self-renewal pathways in myeloid malignancies. Cell Commun Signal 11, 1–1 (2013). 23675967 10.1186/1478-811X-11-33 PMC3665484

[b38] van Noort V. *et al.* Cross-talk between phosphorylation and lysine acetylation in a genome-reduced bacterium. Mol Syst Biol 8, 1–16 (2012). 10.1038/msb.2012.4 PMC3293634 22373819

[b39] Song J. J. Cross-talk between JIP3 and JIP1 during glucose deprivation: SEK1-JNK2 and Akt1 act as mediators. J Biol Chem 280, 26845–26855 (2005). 15911620 10.1074/jbc.M502318200

[b40] Dumont J. E., Dremier S., Pirson I. & Maenhaut C. Cross signaling, cell specificity, and physiology. AJP: Cell Physiology 283, C2–C28 (2002). 12055068 10.1152/ajpcell.00581.2001

[b41] van Noort V. *et al.* Cross-talk between phosphorylation and lysine acetylation in a genome-reduced bacterium. Mol Syst Biol 8, 1–16 (2012). 10.1038/msb.2012.4 PMC3293634 22373819

[b42] Klinke D. J., Cheng N. & Chambers E. Quantifying crosstalk among interferon-γ, Iinterleukin-12, and tumor necrosis factor signaling pathways within a TH1 cell model. Science Signaling 5, ra32–ra32 (2012). 22510470 10.1126/scisignal.2002657 PMC3373264

[b43] Wang C.-C., Cirit M. & Haugh J. M. PI3K-dependent cross-talk interactions converge with Ras as quantifiable inputs integrated by Erk. Mol Syst Biol 5, 246 (2009). 19225459 10.1038/msb.2009.4 PMC2657535

[b44] Junttila M. R., Li S. P. & Westermarck J. Phosphatase-mediated crosstalk between MAPK signaling pathways in the regulation of cell survival. The FASEB Journal 22, 954–965 (2007). 10.1096/fj.06-7859rev 18039929

[b45] Fraser I. D. C. & Germain R. N. Navigating the network: signaling cross-talk in hematopoietic cells. Nat Immunol 10, 327–331 (2009). 19295628 10.1038/ni.1711 PMC2776706

[b46] Tofts P. S. *et al.* Estimating kinetic parameters from dynamic contrast-enhanced T(1)-weighted MRI of a diffusible tracer: standardized quantities and symbols. J Magn Reson Imaging 10, 223–232 (1999). 10508281 10.1002/(sici)1522-2586(199909)10:3<223::aid-jmri2>3.0.co;2-s

[b47] Alon U. An Introduction to systems biology: design principles of biological circuits. Chapman *&* Hall/CRC 1–162 (2007).

[b48] Ito Y., Toyota H., Kaneko K. & Yomo T. How selection affects phenotypic fluctuation. Mol Syst Biol 5, 1–7 (2009). 10.1038/msb.2009.23 PMC2683726 19401676

[b49] Legewie S., Blüthgen N. & Herzel H. Mathematical modeling identifies inhibitors of apoptosis as mediators of positive feedback and bistability. PLoS Comput Biol 2, e120 (2006). 16978046 10.1371/journal.pcbi.0020120 PMC1570177

[b50] Qiao L., Nachbar R. B., Kevrekidis I. G. & Shvartsman S. Y. Bistability and oscillations in the Huang-Ferrell model of MAPK signaling. PLoS Comput Biol 3, e184 (2007). 10.1371/journal.pcbi.0030184 PMC1994985 17907797

[b51] Bhalla U. S. & Iyengar R. Robustness of the bistable behavior of a biological signaling feedback loop. Chaos 11, 221 (2001). 12779455 10.1063/1.1350440

[b52] Spencer S. L., Gaudet S., Albeck J. G., Burke J. M. & Sorger P. K. Non-genetic origins of cell-to-cell variability in TRAIL-induced apoptosis. Nature 459, 428–432 (2009). 19363473 10.1038/nature08012 PMC2858974

[b53] Albeck J. G., Burke J. M., Spencer S. L., Lauffenburger D. A. & Sorger P. K. Modeling a snap-action, variable-delay switch controlling extrinsic cell death. Plos Biol 6, e299 (2008). 10.1371/journal.pbio.0060299 PMC2592357 19053173

[b54] Hornberg J. J., Bruggeman F. J., Westerhoff H. V. & Lankelma J. Cancer: A systems biology disease. Biosystems 83, 81–90 (2006). 16426740 10.1016/j.biosystems.2005.05.014

[b55] Kaneko K. Evolution of Robustness to noise and mutation in gene expression dynamics. PLoS ONE 2, e434 (2007). 17502916 10.1371/journal.pone.0000434 PMC1855988

