# Supplementary material for: Negative Interactions and Feedback Regulations Are Required for Transient Cellular Response
Source: Sci Rep. 2014 Jan 16;4:3718. doi: 10.1038/srep03718 (PMC3893651; doi:10.1038/srep03718)
Supplement: Supplementary Information — S1: Minimal cascade and its reaction details [file srep03718-s1.pdf]

Supplementary Information 1

Negative Interactions and Feedback Regulations Are Required for Transient Cellular Response

Authors: Mohammad Mobashir, Madhusudhan Thati, Berend Isermann, Tilo Beyer, Burkhart Schraven

Minimal cascade and its reaction details:

a Minimal Signaling Cascade

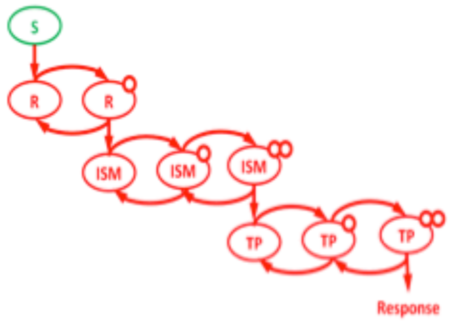

b Minimal Signaling Cascade Reactions

$$R + S \underset{k2}{\overset{k1}{<==>}} Rp + S$$

$$Rp + ISM \underset{k4}{\overset{k3}{<==>}} X1 \overset{k5}{==>} ISMp + Rp$$

$$Rp + ISMp \underset{k7}{\overset{k6}{<==>}} X2 \overset{k8}{==>} ISMpp + Rp$$

$$ISMp + TP \underset{k10}{\overset{k9}{<==>}} X3 \overset{k11}{==>} TPp + ISMp$$

$$ISMpp + TPp \underset{k13}{\overset{k12}{<==>}} X4 \overset{k14}{==>} TPpp + ISMpp$$

$$ISMp + TPp \underset{k16}{\overset{k15}{<==>}} X3 \overset{k17}{==>} TPpp + ISMp$$

Figure S1 (Supplementary data 1): (a) Minimal cascade and (b) the reaction details.
